# Adaptation of problem-solving therapy for primary care to prevent late-life depression in Goa, India: the ‘DIL’ intervention

**DOI:** 10.1080/16549716.2017.1420300

**Published:** 2019-05-20

**Authors:** Amit Dias, Fredric Azariah, Miriam Sequeira, Revathi Krishna, Jennifer Q. Morse, Alex Cohen, Pim Cuijpers, Stewart Anderson, Vikram Patel, Charles F. Reynolds

**Affiliations:** a Goa Medical College, Goa, India; b Sangath, Goa, India; c Graduate Psychology Program, School of Health Sciences, Chatham University, Pittsburgh, PA, USA; d Department of Population Health, London School of Hygiene and Tropical Medicine, London, UK; e Department of Developmental, Neuro-, and Clinical Psychology, Free University of Amsterdam, Amsterdam, Netherlands; f Department of Biostatistics, Graduate School of Public Health, University of Pittsburgh, Pittsburgh, USA, PA; g Department of Global Health and Social Medicine, Harvard Medical School, Boston, MA, USA; h Department of Psychiatry, The University of Pittsburgh School of Medicine, Pittsburgh, PA, USA

**Keywords:** late-life depression, problem-solving therapy, lay health counselors, low- and middle-income countries, indicated depression prevention

## Abstract

**Background**: Depression in late life is a major, yet unrecognized public health problem in low- and middle-income countries (LMICs). The dearth of specialist resources, together with the limited ability of current depression treatments to avert years lived with disability, underscores the need for preventive interventions that can be delivered by lay health workers in primary care settings. We describe the development of an intervention for the indicated prevention of depression in older adults at risk due to subsyndromal symptoms, attending rural and urban public primary care clinics in Goa, India.

**Objectives**: (1) to describe a mixed-methods approach (qualitative and quantitative)to the development of ‘DIL,’ an intervention for preventing the onset of major depression in older adults living with subsyndromal symptoms in Goa, India; (2) to describe resulting components of the ‘DIL’ intervention; and (3) to present data on the feasibility, acceptability, and benefit of DIL to participants.

**Methods**: We followed a mixed-methods design, including in-depth interviews, focus group discussions, a theory of change workshop to develop a logic model, and an open-case series.

**Results**: The mixed-method approach led to the development and adaptation of the DIL (Depression in Later Life) intervention for the indicated prevention of depression in older adults. The intervention was delivered by lay health counselors (LHCs). ‘DIL’ is a hybrid model of simple behavioral strategies grounded in Problem-solving Therapy for Primary Care, improved self-management of common, co-occurring medical disorders such as diabetes mellitus, and pragmatic assistance in navigating to needed social services. The use of ‘DIL’ in an open-case series with 19 participants led to a moderate reduction in symptoms of depression and anxiety on the General Health Questionnaire. A pictorial flipchart was developed to assist in delivering the intervention to participants with low levels of literacy. High rates of participant retention and satisfaction were achieved.

**Conclusion**: The DIL intervention was adapted to the local context for delivery by lay health counselors and was found to be acceptable and feasible among the elderly participants in the study.

## Background

Depression is projected to be one of the three leading causes of disease burden in 2030. Specifically, depression in older adults is associated with significantly increased health care use [1] and economic costs [2]. Major depression is more often than not comorbid with other chronic conditions, significantly increasing the disability associated with these conditions and worsening family caregiver burden [3]. Depression is associated with worse physical health outcomes and poor treatment adherence [4]. Depression in older adults often goes unrecognized and undiagnosed due to lack of awareness and the failure to screen, together with the varied clinical presentations of depression in the elderly population compared with the adult population [5].

The World Health Organization (WHO) estimates that low- and middle-income countries (LMICs) carry about 70% of the total burden of mental disorders, whereas high-income countries enjoy about 90% of the global mental health resources [6]. About two thirds of the people in LMICs with serious mental illness have no access to any care [7]. The prevalence of late-life depression in community samples in India ranges from 8.9% to 62.16%, with a median prevalence of 18.2%. This is considerably higher than the global median rate of 5.4% [8]. When one considers the prevalent treatment gap together with unique characteristics of late-life depression, the treatment gap may be even wider for this population.

In the face of the treatment gap and dearth of resources in LMICs, task sharing or task shifting has become increasingly important. Task sharing/shifting can be defined as ‘a process that ensures the transfer of specific tasks from highly qualified specialists to other health workers with less expertise (or qualifications) in a specified area of competence – in this case mental illness’ [9]. The MANAS trial in India employed a stepped-care approach to task sharing and demonstrated the effectiveness of using lay health counselors to identify and treat common mental disorders like depression in primary care [10]. Similarly, in Pakistan, a cognitive behavior therapy based intervention delivered by lay health workers for mothers with perinatal depression proved to be effective compared with usual care [11]. Task sharing has been used successfully in other parts of the developing world as a way to combat limited availability of ‘trained human resources’ [4,12]. It has been proven to be an effective approach to implementing health care models in LMICs such as India, e.g. [13–15], 2014 [10,16].

We describe in this paper an intervention development project (‘DIL’ – Depression in Late Life) aimed at developing and adapting a feasible and scalable intervention based on problem-solving therapy to prevent depression in later life and thereby promote well-being. ‘DIL’ means ‘heart’ in Hindi (National language in India). In this study, we tried to answer three questions: What is the current situation for older people at risk for depression? What intervention should be used to prevent depression? How should the intervention be delivered?

The study was conducted by Department of Preventive and Social Medicine of the Goa Medical College, in partnership with Sangath, in both an urban and a rural setting in Goa, India. This project was supported by the National Institute of Mental Health, USA (R34 MH99667) and is a collaboration between the University of Pittsburgh, USA and the London School of Hygiene and Tropical Medicine, UK. We registered the study with Clinicaltrials.gov.

## Methodology

The study followed the principles of a mixed-methods design. These methods comprised a literature review, in-depth interviews with key stake holders, theory of change workshops, focus-group discussions, and an open-case series. The results were triangulated to inform the development of the DIL intervention and to optimize the likelihood of participant enrolment and retention, and effectiveness in preventing depression in the elderly.

### Literature review

Our literature review revealed that prevention of depression in later life is important particularly in LMICs [3]. Problem-solving Therapy (PST) [17] and Brief Behavioral Treatment for Insomnia (BBTI) [18] were found to be effective therapies that were used in the high-income countries to prevent depression [3]. We examined peer-reviewed literature on the prevention of depression and associated anxiety disorders, building upon the MANAS study [10], and formative research for the Premium Study [19] on depression, which included an intensive literature review as one of its components. This procedure enabled us to address evidence from low-resource settings, causal understanding, and help-seeking in our local context. In addition, this process enabled us to map out strategies for preventing depression and anxiety; to identify potential barriers for implementation and strategies to resolve them; and to identify the minimum skills required to deliver these components. Similar to the conduct of the MANAS trial [10], DIL tested the effectiveness of using Lay Healthy Counselors. However, the focus of DIL was on prevention, rather than treatment of prevalent depression, with a specific focus on older adults rather than a mixed-aged sample. DIL’s intervention model was also a hybrid of three distinct strategies, as detailed below (PST, education in self-management of co-occurring medical disorders, and navigational assistance to needed social and financial resources [20].

### In-depth interviews

In-depth interviews (IDI) were conducted with patients above the age of 60 attending the rural health and training center at Mandur (*n* = 10) and the urban health center at St Cruz (*n* = 10) (Table 1). We used purposive sampling to recruit participants for IDI. Eligible participants were approached, and consent was sought for participating in the IDI. We recruited equal numbers of men and women and in two age group (60–70 years/71 and above). The IDI was conducted primarily at the public health clinic, with complete privacy, and in some cases at the participant’s home. IDI lasted for 45–60 minutes and were audio-recorded after obtaining consent from the participants. These tapes were then transcribed verbatim and translated into English. The guide was developed after extensive discussions during fortnightly conference calls of DIL’s steering committee (i.e. the authors of this report), and consensus was obtained. The guide was then translated to Konkani (local language) and back-translated to English to verify the validity of the guide. The interview was predominantly conducted in Konkani. We developed a coding framework via workshop-based consensus. The transcripts of the IDI were then coded by two independent reviewers and matched for agreement. Coding was then analyzed using a qualitative data analysis software (MaxQDA).

**Table 1.** In-depth interviews.

Participants	No. of IDI	
Elderly participants	20	(m = 10; f = 10)
Depressed participants	5	(m = 0; f = 5)
Caregivers	5	(m = 2; f = 3)
Experts	2	(m = 1; f = 1)
Total	32	(m = 13; f = 19)

We also conducted IDI with depressed elderly participants (*n* = 5) and their carers (*n* = 5) in order to obtain deeper insights about depression and its prevention in the elderly population. We modeled a purposive sampling approach to recruit participants. We referred to the medical records maintained at the rural health center to provide us with the list of participants who had been clinically diagnosed with depression and in treatment. These individuals were then approached for participation in IDI. We also conducted IDI with their caregivers (person who was immediately looking after the person with depression) to understand their perspectives. Strict measures were taken to maintain privacy during the interview. The interviews were audio-recorded following consent and were transcribed verbatim and translated into English.

Finally, to obtain key information related to areas such as government schemes, NGO involvement in elderly care, and Old Age Home quality and accessibility, we conducted IDI on two local experts involved in care of the elderly. The content of the IDI and their emerging themes were discussed by the DIL steering committee during fortnightly conference calls. In addition, we conducted IDI among participants in the final confirmatory trial to determine replicability of early and late findings. In this manner, we triangulated observations made during initial IDI, theory of change workshops, and recurrent themes emerging in our formative pilot work of the open-case series.

### Theory-of-change (ToC) workshop

**Figure 1.**
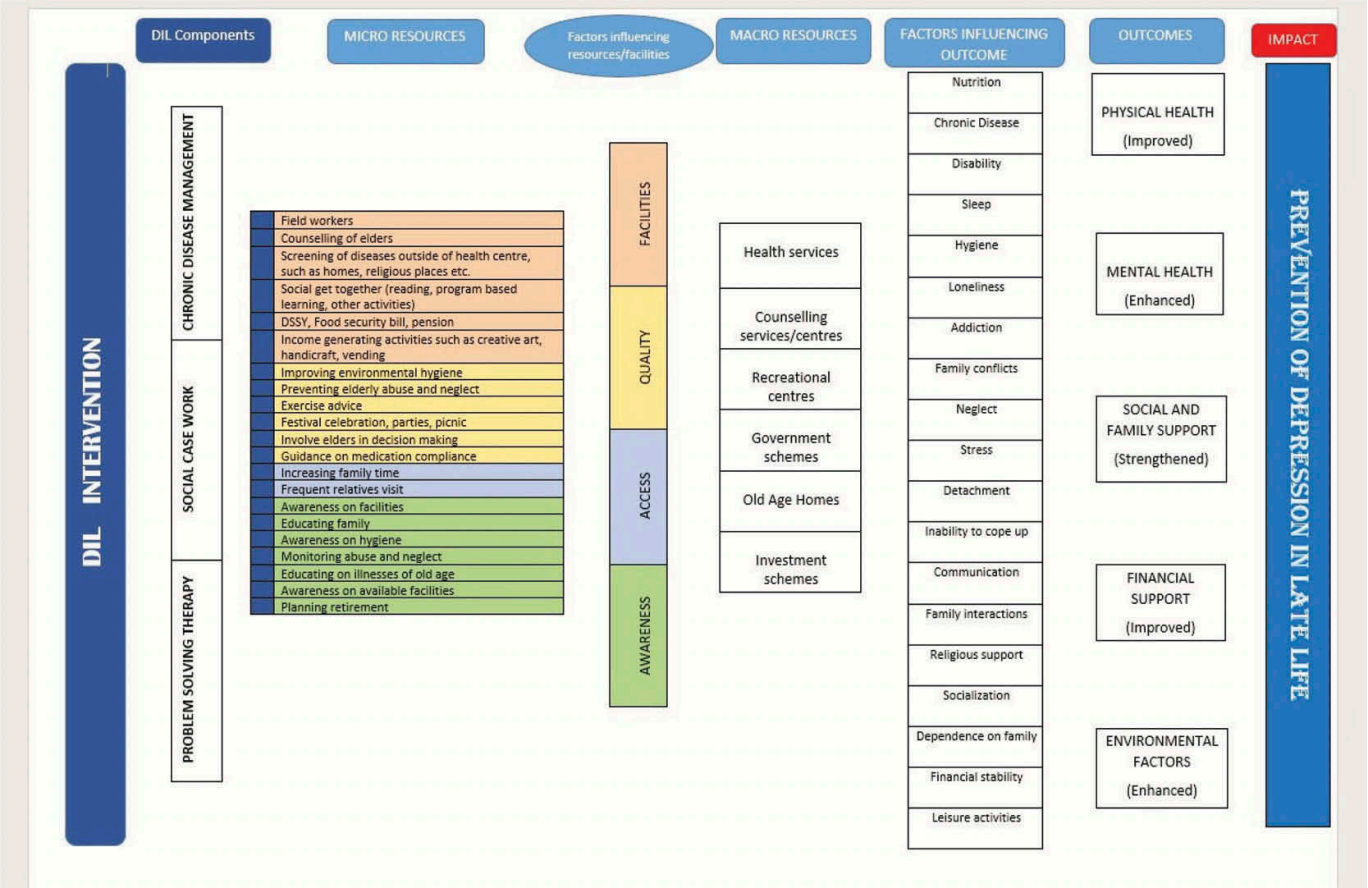
Theory-of-change pathway.

We conducted a ToC workshop with key stakeholders for mapping out a logical change pathway needed to achieve the objectives of the DIL study. A total of 27 participants were split into five groups. The stakeholders included nursing staff, community health workers, coordinators, social workers, resident medical doctors, medical interns, and psychologists. The participants engaged actively, and a ToC framework (Figure 1) was developed. The use of focus groups with key stakeholders and theory of change workshops allowed us to form a logic model for the DIL intervention research, specifying stakeholders’ understanding of the current situation, the changes they hoped to bring about through the program with and for whom, the activities planned to contribute toward this change, the resources needed to be put into the effort, assumptions they are making, and external events that could influence results, needs of key stakeholders, resources available, acceptable methods of implementation, and formative and summative outcomes.

### Case series

To test the acceptability and feasibility of delivering the intervention with the help of lay health counselors, we conducted an open-case series at a rural site in northern Goa (Curca). We targeted recruitment of 20 participants based on the eligibility criteria designed for the study. The instruments used for evaluation were MINI 6.0 [21], General Health Questionnaire [22], WHO Disability Adjustment Schedule-12, and Hindi Mini Mental State Examination.

The inclusion criteria stipulated age above 60 years, residence in the catchment area, fluency in English, Hindi, or Konkani, GHQ score 4 or above, negative on MINI 6.0, and HMMSE score >24 (in order to include subthreshold depression and to rule out clinical depression and cognitive impairment). Participants currently on antidepressant medications, having moderate to high suicidality, or diagnosed with dementia or with terminal illnesses, were excluded from the study. Following consent, participants were screened by the researchers at the public health clinic or at homes to confirm eligibility. If eligible, researchers sought informed consent for participation in the case series. GHQ, MINI 6.0, WHODAS 2.0 and self-reported chronic disease questionnaires were used at baseline. The lay health counselors visited all the participants in the case series to deliver the intervention. Following the intervention, independent researchers conducted the outcome evaluation.

There were 21 participants in the open-case series (mean age 69, 19 women, 20 Hindus, 17 illiterate or with no formal education, 16 living with their adult children, and 18 without a spouse). The most prevalent co-occurring medical disorders were hypertension (13/21), diabetes (6/21), heart disease (5/21), arthritis (5/21), and COPD/asthma (5/21). Of the 21 participants enrolled, 19 completed the feasibility trial embedded in the open-case series. None required referral to a mental health specialist.

DIL employed four lay health counselors: three women and one man, with an age range of 35–45, and generally with a college level of education but with no prior mental health training or experience. Lay health counselors were chosen for their interest in working with older adults.

### Exit IDI with participants in the open-case series

Following the case series, exit IDI were conducted in nine participants. The guide focused on the intervention content, delivery, participant satisfaction, and overall usefulness of the program. Sections of the guide also included suggestions and recommendations to improve the program.

We recruited a range of participants whose GHQ scores had improved (*n* = 5), remained constant (*n* = 2), or worsened (n = 2) following the intervention, to gain a broad and unbiased view of the DIL program as implemented in the case series.

### Focus-group discussion (FGD)

An FGD discussion was conducted with the researchers and lay health counselors (*n* = 6) who were involved in the case series. The aim of the FGD guide was to evaluate the acceptability and feasibility of the intervention and to understand the experiences of the researchers and counselors during the case series. The duration of the FGD was approximately 90 minutes.

## Results

We used a mixed-methods approach to develop and to adapt the intervention and its delivery by lay health counselors. Every stage of the intervention development process was closely monitored by the investigators on site in Goa and during fortnightly conference calls with other members of the investigative team based in the US and in Europe. The entire team also convened on site in Goa annually and held semiannual teleconference calls with a multinational Data Safety Monitoring Board over the three year period of the study.

**Figure 2.**
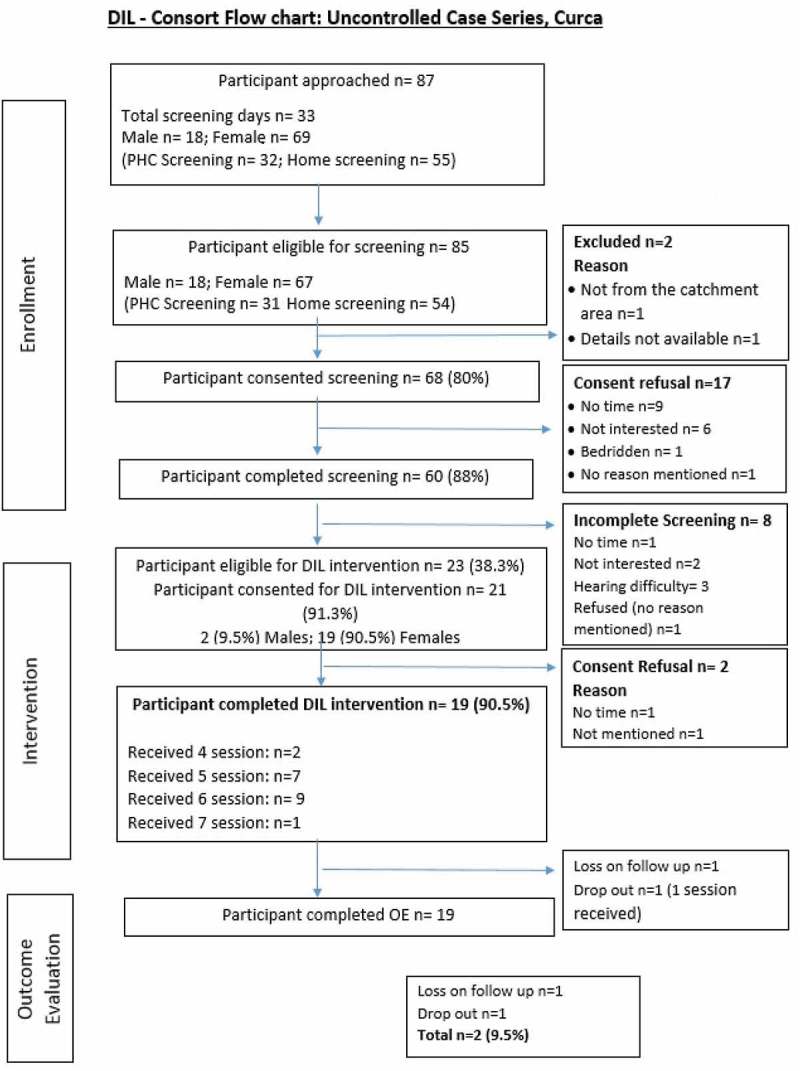
Consort – case series.

For the open-case series, we approached 87 (18M, 69F) participants (Figure 2) over 33 working days. Of these participants, 68 consented to be screened and 60 completed the screening. Of the 23 who were eligible for the study, 21 (19F, 2M) participants consented. The intervention was delivered to 19 participants, with two dropping out from the study. Outcome evaluation (*n* = 19) was done after 3 months using the same questionnaire. The mean GHQ score at baseline was 5.4 (1.5) (95% CI: 4.7–6.1) and at outcome was 3.3 (2.0) (95% CI: 2.4–4.3). The mean WHODAS score at baseline was 21.7 (8.9) (95% CI: 17.7–25.8 and at outcome was19.4 (7.1) (95% CI: 16.0–22.9). GHQ change scores demonstrated a moderate effect size that was clinically meaningful in reducing the key proximal risk factor for major depression (namely, subsyndromal symptoms of depression). Quality of life and functional status also improved.

Qualitative analyses of IDI with participants showed that they preferred the use of the word ‘tension’ rather than ‘depression.’ Many endorsed worries regarding an uncertain future related to conflicted relationships with their grown children and to deteriorating health. Participants endorsed the sessions as enjoyable. Because of the low levels of literacy among DIL participants, we found it essential to use flip charts illustrating the strategies of PST and of BBTI. Figures found to be particularly helpful include those illustrating the ‘Stress Cycle,’ the ‘Upward Spiral,’ and ‘Mood Rating Scale.’ Lay health counselors contributed meaningfully to the development of illustrative flip charts and to the development of engagement and retention strategies.

As elaborated below, the four lay health counselors participated in conference calls with PST supervision every two weeks. PST supervision centered on case-based presentations and discussions about adapting PST to local conditions for use in participants with low literacy levels and with difficulty recalling the seven steps of traditional PST as developed in high-income countries.

### Adaptations to content and delivery of the DIL intervention

Based on our literature review, we learned that PST is effective in the treatment of prevalent mental disorders like depression [16]. PST is a brief and focused psychological and behavioral intervention that has been used with a variety of groups including people with depression, chronic illness, and suicidal thoughts and behaviors. In essence, PST is a learning-based psychotherapy that employs strategies of behavioral activation. It aims to teach a positive problem-solving orientation, to improve active coping, and to enhance a sense of self-efficacy. We adapted the PST model used in high-income countries as the basis for the DIL intervention. Our efforts were informed by research into PST’s effects on executive impairment in older depressed adults, such as work from the Alexopoulos laboratory [23].

For simplicity of administration, we divided the DIL intervention into three phases: engagement, problem-solving, and termination (‘ending well’). Each phase had a specific set of tasks and goals that needed to be met before moving on to the next phase. For example, the main goal of phase 1 (engagement) was rapport-building, introducing the DIL program to the participant, explaining the DIL brochure, and addressing queries about the program. The main goal of phase 2 was to teach problem-solving skills; tasks revolved around helping participants to apply these skills to current problems and those that might arise in the future. Generalizing the skills learned was the main goal of the third phase (‘ending well’).

**Figure 3.**
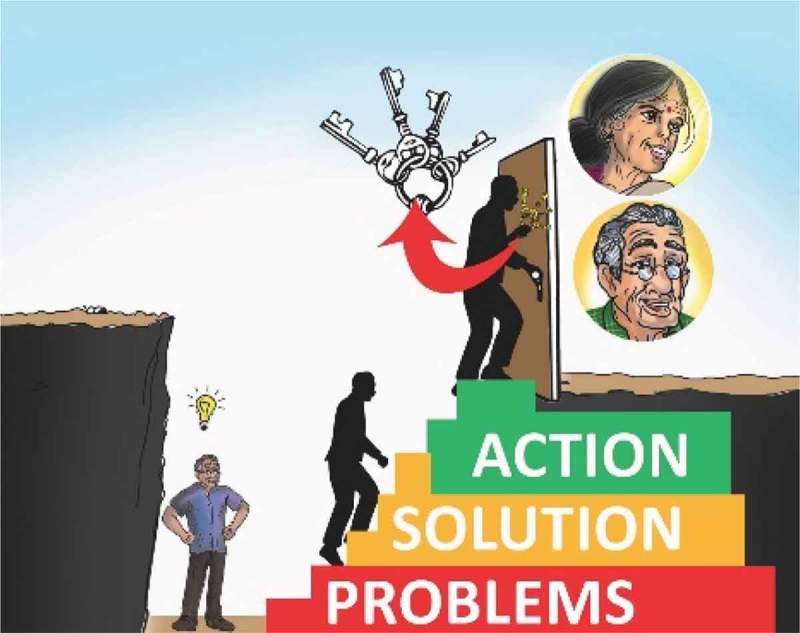
Use of illustration as a visual aid to teach PST in three steps.

The most significant change in content of the intervention was reduction of the seven steps of PST into three main steps, in order to facilitate easy recall and application by older adults. During the first phase of adaptation, the seven steps of PST were taught with the help of an acronym INSPIRE (**I**dentify the problem, **N**ote a realistic achievable goal, **S**earch for all possible solutions, **P**ros and cons, **I**dentify the preferred solution, **R**un with it, **E**xamine the result). However, during the in-depth exit interviews following the open-case series, eight out of nine (88.9%) participants were unable to recollect the steps. The findings from the FGDs suggested the need for a simpler, more culturally relevant reminder. Hence, we simplified the PST’s seven steps into a three-step process, namely: Problem, Solution, Action. In the local Konkani language, these three steps were translated to form the acronym ‘SAUD,’ which means ‘health.’ We also developed a flip chart of illustrations to assist lay health counselors in training participants. The illustrations depicted an upward spiral as a man climbing up three steps and attempting to open a door with a bunch of keys. Each step used a different color for further simplification and to emphasize that each step is distinct from the other (Figure 3).

We noted that use of pictures during the session helped participants remember the steps of problem-solving and active coping. Since our goal was development of an intervention effective in preventing depression, it was important for participants to remember the steps of PST in order to address future problems. With this in mind, a flipchart with figures illustrating the core principles and ideas of the intervention was developed as a visual aid for training. The DIL flipchart is a calendar layout document with pictorial representation of the intervention ideas on one side and the text explanation of the picture on the opposite side. The pictures were used to engage participants while the text reminded the counselors about the key points to address. The DIL flipchart covers topics such as a mood rating scale, the seven steps of PST, a summary sheet highlighting the three abridged steps of PST, sleep hygiene, hypertension, diabetes and heart disease management, exercises for knee and lower back aches, and finally substance-abuse-related information. These were to be used as and when required, depending on the health needs of the participants. For example, we would only turn to the diabetes page of the flipchart for participants living with diabetes.

Other tools developed included a set of worksheets for participants to monitor their sleep behavior, engagement in pleasurable activities, a diabetes recording chart, and a brochure highlighting key learning points from the intervention.

Our qualitative work and the open-case series revealed a need for helping participants access needed resources, for example, by applying for government financial assistance when the participant could not write or by accompanying him/her to the doctor when there was lack of support. We designated this component as ‘social case work’ but made it clear that the lay health counselor would not unilaterally solve the problem but would help and motivate participants to find a solution to for problems and help with implementation of solutions.

Sessions typically lasted 30–40 minutes. Participants grew weary after 40 minutes, affecting their concentration and engagement. Initial sessions in the case series were scheduled one week apart, but frequency was modified to two weeks on request of participants. A courtesy call was made to participants when the gap between sessions extended beyond the schedule. The intervention was delivered in the participant’s choice of location. Participants who wanted to involve their significant other (SO) in the sessions were supported in doing so.

To optimize feasibility and to accommodate low literacy levels, we reduced written homework and assignments significantly as compared with the original version of PST. Worksheets were typically completed by the lay health counselor in the presence of the participant. In cases where a carer or SO was literate, he/she was encouraged to fill the PST worksheets for the elder. Regardless of how the PST worksheet was filled out, lay health counselors facilitated independent problem-solving by encouraging elders to be actively involved in each step of the model and to take increasing ownership of the problem-solving process, analogous to the process of encouraging clients to begin to write the text in their own PST worksheets in high-income countries.

Participants in the open-case series were enrolled for one year. We incorporated two booster sessions, one at seven months and a second at ten months. The aim of booster sessions was to strengthen the skills which the participant had difficulty learning and to ensure continued practise. The structure of booster sessions is the same as that of the initial sessions.

**Figure 4.**
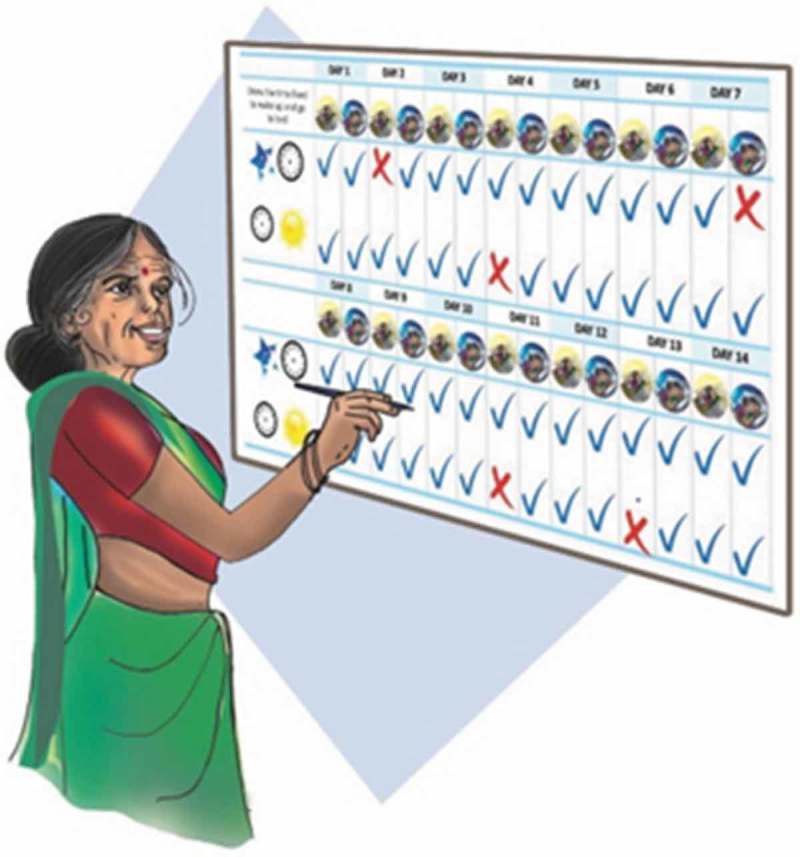
Sleep-monitoring chart.

**Figure 5.**
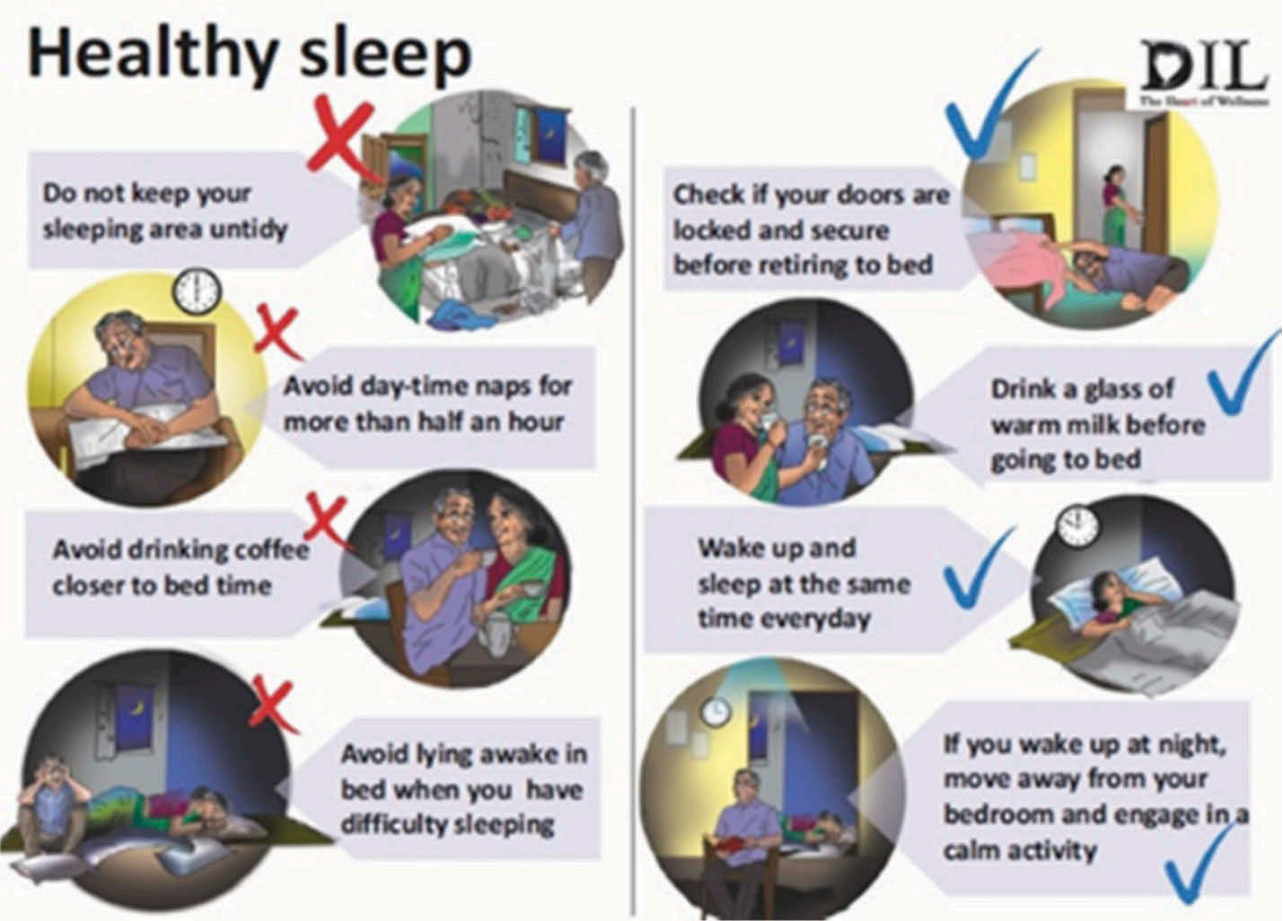
Sleep hygiene (dos and don’ts).

In order to carry out education and monitoring components of BBTI, we used clinical interviews to understand the participant’s sleep problem rather than standardized self-report questionnaires and sleep dairies. The new sleep-wake schedule was collaboratively discussed based on the clinical interview and a single-sheet sleep monitoring chart was provided to the elder. The elder was taught to put a tick mark if the new sleep-wake routine was followed each day and record his/her mood beside it (Figure 4). The counselor would review the chart in the following session. Sleep hygiene was explained to DIL participants in the form of illustrations of dos and don’ts (Figure 5).

### Illustration to support BBTI and training in sleep hygiene

### Delivery of the intervention

During first phase of engagement, lay health counselors encouraged participants to talk about signs and symptoms of depression by using simple illustrations. Similarly, the counselor explained the mood rating scale and the significance of identifying one’s mood and engaging in pleasurable activities at this point. They would then talk about the stress cycle, together with downward and upward spirals. All psycho-education was done in the context of a conversation using the illustrations, with the goal of creating a problem-solving and active-coping orientation in the participants.

Following engagement (step 1), counselors proceeded to teaching problem-solving. During this phase, counselors explained all seven steps of PST to the participant. Each step had a separate illustration and was shown on a separate page. After the seven steps were explained, the abridged version of three steps was pictorially presented as a summary. Elders who could recall all seven steps were encouraged to do so. Others were encouraged to practise the three-step approach while problem-solving. After talking about examples in the flipchart, counselors would encourage participants to apply the seven steps to a personal problem. Counselors always followed the seven-step approach while problem-solving during the session. Some common problems selected by participants included sleep maintenance insomnia such as awakening in the middle of the night, and chronic illnesses such as difficulty managing hypertension, diabetes, arthritis, and need for cataract surgery. Lack of pleasurable activities was also seen by participants as a common problem.

The termination or ‘ending well’ phase (step 3) focused on teaching participants how to generalize problem-solving skills to other problems in their lives. The sessions in this phase were similar to those in step 2, with the only distinction being that the elder was encouraged to do most of the problem-solving with minimal prompting from the counselor. Counselors would facilitate a healthy termination of the counseling relationship by highlighting participant progress in active coping and in an increased sense of confidence in solving problems (self-efficacy).

A chart was used to monitor participant engagement in pleasurable activities. In each case, counselors would emphasize the relationship between pleasurable activity and improvements in mood. A diabetes record was also given to those with diabetes that contained a table to chart their blood sugar levels whenever they visited the doctor. This sheet also had pictorial representations of strategies for the management of diabetes.

Because we were dealing with a vulnerable population with a risk of suicide, we developed and piloted a protocol for early detection and management of suicide risk. Participants who continued to report a mood rating of 4 and 5 were asked about suicidal ideation, using item 9 from the Patient Health Questionnaire 9 (PHQ) questionnaire [17]. This item assessed suicidal thoughts and behavior of the participant. A positive response to this item would lead to a detailed suicide risk assessment by the counselor. The suicidality scale of the MINI was used to obtain a quantitative measure of level of risk for suicide. Counselors were trained to identify both risk and protective factors. Those participants in medium- and high-risk category were referred to a psychiatrist in the primary health center. Participants at low risk were given a suicide-prevention brochure and provided with counseling to highlight their protective factors.

Elders who missed three scheduled appointments or were not contactable for one month were declared as ‘unplanned discharges’. Those who had a severe adverse event and needed to be referred to a specialist were also deemed to have unplanned discharges. Participants who completed the required number of sessions as planned were declared as ‘planned discharges’.

Participants perceived the DIL intervention to be helpful. Some key mediators associated with better outcomes included problem-solving, behavioral activation, self-monitoring of mood, psycho-education (enhanced by figures in the flip chart), and the supportive skills of the lay health counselors. We also noted that participants continued to practice skills in their daily lives, as encouraged by the use of a booster session.

### Supervision and fidelity

Supervisory meetings were held every two weeks via Skype and lasted 30–45 minutes each. An agenda was circulated one day prior, along with a detailed report of the minutes of the previous supervision. The agenda usually consisted of the general intervention updates as reflected in a consort chart, challenges faced while delivering the intervention, tools developed to aid intervention delivery, and scheduled date for the next call. The challenges faced and strategies to overcome them (Table 2) were discussed during these meetings. In addition to these fortnightly supervisory meetings, local supervision meetings were held once a week with local intervention supervisors and lay health counselors. During the weekly supervision meetings, one audio-recorded session was played. The group consisted of all four lay health counselors and two supervisors, who would provide feedback to counselors and complete the DIL Therapy Quality Assessment Scale.

### Competency assessment

Counselors were tested for competency in delivering the intervention using a standardized competency assessment tool modified from ‘The Thinking Healthy Program’ (TTHP) [24]). The competency assessment process consisted of a case vignette presented to one counselor at a time and an actor. The actor was provided with a script detailing standard responses to the counseling interview. The counselor was presented with a list of tasks to be accomplished in a period of 10 minutes of role play with the actor. Each counselor was presented with a different case vignette and given 15 minutes to prepare for the task. These tasks usually involved identifying the participant’s main stressor and going through one of the key principles of the intervention with the elder. The competency scale had two sections – counseling-related skills and DIL-content related skills. Each section had prerequisite skills to be demonstrated and actions that were not to be demonstrated. An example of a skill to be demonstrated is ‘summarizes what the elder says’; an example for an action not to be demonstrated is ‘impose advice or adopt a bossy demeanour’. Counselors were given one point for each skill demonstrated. In order to clear the competency test, a counselor needed to obtain an overall 80% score on the total skills to be demonstrated. The detailed scoring of the competency assessment scale is shown in Table 3. Counselors who cleared the competency test were assigned a caseload of the trial participants.

**Table 2.** Challenges and strategies used during open-case series.

Challenges faced	How they were handled
Elder would not generate solutions on his own during the session and wanted the counselor to give him advice	Elder was reminded that solutions that work for one person may not work for another, and that it is best for each person to come up with their own solutions
Elders had difficulty recalling the content of the previous session	Counselor summarized and gave the elder probes to recall the action plan
Family did not think that their elder needed counseling	Benefits of the program, such as chronic illness management, better sleep, and improved pleasurable activities, were explained to the family. Did not use the term ‘depression’ while talking to the family and the participants.
Interruptions from family members and neighbors during the home-based sessions	Session was resumed with a brief summary after informing the interrupter about the need for privacy
challenges related to scheduling sessions: missed appointments not reachable on the telephone busy with house work renovation of house – too much noise	Appointment was confirmed via telephone (when available) in the morning and only then visit the elder. When no telephone, multiple visits to the elder’s house were carried out. Flexible gaps between sessions provided.
Challenges related to duration of sessions – elders vent out and session goes beyond 60 minutes	give space to the elder to cry use reflection begin the next session by retelling the elder his/her story

**Table 3.** Competency assessment final scoring.

For section 1, a counselor should demonstrate:	(i) A minimum of four of the skills to acquire and maintain competency(ii) Among the skills that should not be present in a session, at least two of them should not be demonstrated
For section 2, a counselor should demonstrate:	(iii) A minimum of five of the core skills

We used the results of the open-case series to inform the conduct of a larger confirmatory clinical trial in indicated prevention of depression. We have continued to demonstrate acceptable recruitment feasibility by meeting 100% of the targeted randomization (*n* = 181), with fewer than 20% of eligible subjects refusing randomization and with high retention (85%).

### Ethics and protection of participants’ privacy

Ethical clearance was obtained from the ethics committees at Goa Medical College, Sangath, Indian Council of Medical Research, and from the institutional review boards at the London School of Hygiene and Tropical Medicine and the University of Pittsburgh. Participants were provided with comprehensive information about the study, and family members of illiterate participants were asked to sign as witnesses to the consent procedure after the information was read out aloud to them. Thumb impressions of the illiterate participants were also taken. Informed consent was obtained from all participants prior to conducting any data collection. Hard copies of the data collected were secured in locked cabinets, and participants were referred to by trial IDs during all team discussions to protect their privacy. The soft copies were secured with passwords and accessible only to the data manager.

## Discussion

This paper describes the first effort to systematically adapt PST for delivery by lay health counselors in an LMIC and to test its feasibility and acceptability in the local context for the prevention of depression in late life. Our findings suggest that the DIL model, adapted from PST and delivered by lay health counselors with relatively low intensity, is not only feasible but also acceptable to the local population.

In principle, the adapted intervention is based on the same theoretical construct as the original PST. However, the resulting DIL intervention required adaptation in some aspects of its content and delivery to enhance contextual acceptability and to enable the intervention to be delivered by lay health counselors to older adults with limited literacy.

The potential impact of DIL derives from its novel focus on prevention of mental disorders in later life in LMICs, its use of lay health counselors to support scalability via the use of task shifting and sharing, and the use of a combination of synergistic approaches encompassing training in problem-solving, BBTI, education in self-management of chronic diseases, and pragmatic case management to facilitate access to needed financial and psychosocial resources.

Going forward, we envision three key foci for future research: (1) identification of bio- and psychosocial risk markers, (2) expansion of outcomes to include measures of physical and neurological health (including cognitive status), and (3) enhancement of external validity via the use of large cluster-randomized trials. We anticipate that markers of risk may help identify which persons in particular may benefit from the use of preventive strategies, thereby optimizing the efficient use of scarce mental health resources in LMICs. Biomarkers may also serve as moderators of intervention response and may say something about how interventions work to reduce the risk for depression and other common mental disorders.

## Paper context

Preventing depression in vulnerable older adults is a matter of increasing global health priority. In order to address the shortage of mental health specialty expertise in low- and middle-income countries, we have developed an approach to depression prevention (called ‘DIL’) that can be delivered by lay health counselors to older adults attending rural and urban primary care clinics. We describe in this paper the adaptation of problem-solving therapy in primary care (PST-PC) for the indicated prevention of major depression, and we present the results of an open-case series documenting its feasibility, acceptability, and benefit.
